# Population segments as a tool for health care performance reporting: an exploratory study in the Canadian province of British Columbia

**DOI:** 10.1186/s12875-020-01141-w

**Published:** 2020-05-31

**Authors:** Julia M. Langton, Sabrina T. Wong, Fred Burge, Alexandra Choi, Niloufar Ghaseminejad-Tafreshi, Sharon Johnston, Alan Katz, Ruth Lavergne, Dawn Mooney, Sandra Peterson, Kimberlyn McGrail

**Affiliations:** 1 Centre for Health Services and Policy Research, The University of British Columbia (UBC), 201-2206 East Mall, Vancouver, BC V6T 1Z3 Canada; 2 School of Nursing, UBC, Vancouver, Canada; 3 Department of Family Medicine, Dalhousie University, Halifax, NS Canada; 4 Department of Family Medicine, University of Ottawa, Ottawa, ON Canada; 5 Department of Family Medicine and Community Health Sciences, University of Manitoba, Winnipeg, MB Canada; 6 Faculty of Health Science, Simon Fraser University, Burnaby, BC Canada; 7 School of Population and Public Health, UBC, Vancouver, BC Canada

**Keywords:** Primary care, Performance measurement, Population segmentation, Risk adjustment, Health care costs, Administrative data

## Abstract

**Background:**

Primary care serves all age groups and individuals with health states ranging from those with no chronic conditions to those who are medically complex, or frail and approaching the end of life. For information to be actionable and guide planning, there must be some population disaggregation based on differences in expected needs for care. Promising approaches to segmentation in primary care reflect both the breadth and severity of health states, the types and amounts of health care utilization that are expected, and the roles of the primary care provider. The purpose of this study was to assess population segmentation as a tool to create distinct patient groups for use in primary care performance reporting.

**Methods:**

This cross-sectional study used administrative data (patient characteristics, physician and hospital billings, prescription medicines data, emergency department visits) to classify the population of British Columbia (BC), Canada into one of four population segments: low need, multiple morbidities, medically complex, and frail. Each segment was further classified using socioeconomic status (SES) as a proxy for patient vulnerability. Regression analyses were used to examine predictors of health care use, costs and selected measures of primary care attributes (access, continuity, coordination) by segment.

**Results:**

Average annual health care costs increased from the low need ($ 1460) to frail segment ($10,798). Differences in primary care cost by segment only emerged when attributes of primary care were included in regression models: accessing primary care outside business hours and discontinuous primary care (≥5 different GP’s in a given year) were associated with higher health care costs across all segments and higher continuity of care was associated with lower costs in the frail segment (cost ratio = 0.61). Additionally, low SES was associated with higher costs across all segments, but the difference was largest in the medically complex group (cost ratio = 1.11).

**Conclusions:**

Population segments based on expected need for care can support primary care measurement and reporting by identifying nuances which may be lost when all patients are grouped together. Our findings demonstrate that variables such as SES and use of regression analyses can further enhance the usefulness of segments for performance measurement and reporting.

## Background

Routine measurement and reporting can be used to monitor system performance, understand the impact of health care initiatives, identify priorities, and influence health care reform [1–4]. Challenges for primary care performance measurement and reporting include the heterogeneity of patient populations, range of interventions, and intersections with other parts of the health care system [5, 6]. Primary care serves all age groups and individuals with health states ranging from those with no chronic conditions (who require mostly preventive or episodic care) to those who are medically complex, or frail and approaching the end of life. If information is to be actionable and guide planning and evaluation, there must be some population disaggregation based on differences in expected needs for care.

Segmenting populations based on age or discrete diseases is likely to be insufficient in primary care settings, as such groupings still reflect significant heterogeneity [7]. For instance, two patients living with Congestive Heart Failure (CHF) may have different health care needs because of CHF severity, other comorbid health conditions, and/or complex social circumstances. Similarly, segmenting approaches based solely on high health care costs [8, 9] may be limited in the primary care setting as health care costs are typically driven by hospital care and two patients with the same health care expenditures will not necessarily share the same needs from primary care.

Promising approaches to segmentation in primary care reflect both the breadth and severity of health states, the types and amounts of health care utilization that are expected, and the roles of the primary care provider [10]. Segments should be tailored to the needs of information users including patients, providers, and decision makers [11–14], and encompass social determinants of health or vulnerability to enable measurements of health equity given that vulnerable segments of the population have different health care needs compared with the general population [15]. Few studies have incorporated vulnerability into population segments [13, 16] likely because of the complexity and evolving understanding of this construct, and because of the limits of routinely available data to measure it [17]. Using such segments for performance reporting enables comparisons of primary care [6, 10, 14, 18] and quality improvement and/or service planning for particular population sub-groups who stand to benefit most [8, 19–21].

The objective of this paper is to add to the developing literature in this area by assessing population segmentation as a tool to create distinct patient groups for use in primary care performance reporting and ultimately quality improvement. Segments that create distinct patient groups in terms of health care needs (overall and for primary care) can be used to support both learning and improvement within practices and health policy planning and decision making. We implemented principles of regional-level primary care performance measurement [18] to develop and test four population segments that reflect low need, multiple morbidity, medical complexity, and frailty. We further segmented the four groups by the best-available measure of socio-economic status (SES) in an attempt to capture some aspects of vulnerability in relation to socioeconomic context. Finally, we selected three exemplar measures that reflect foundational principles of primary care (access, continuity, and coordination) [22] to explore the variability within and across segments and SES stratification, and assess the potential utility of population segments for reporting on primary care performance.

## Methods

### Setting and population

A cross-sectional observational study using administrate data in the province of British Columbia (BC), Canada which has universal coverage for physician services as determined by the *Canada Health Act* [23]. BC has a population of ~ 4.5 million and the study included all residents meeting the following criteria:

1. ≥18 years as of April 1, 2015
2. Valid records for sex, geographical location [as measured using Local Health Area (LHA [24])] and SES
3. Enrolled in the province’s single-payer Medical Services Plan (MSP) for > 75% of days in each of 2013/14, 2014/15 and 2015/16 noting that the Canadian fiscal year is April 1 to March 31.

Note: ~ 4% of BC residents were excluded because they did not meet criterion 2 or 3.

### Data sources

Administrative data were accessed through Population Data BC [25]. De-identified administrative data files were used to extract data about patient characteristics (consolidation file) [26], physician billings (MSP file) [27], hospital billings (Discharge Abstracts Database, DAD) [28], emergency department visits (National Ambulatory Care Reporting System, NACRS) [29] and medication dispensing (PharmaNet) [30]. For more information about datasets see PopData BC https://www.popdata.bc.ca/data [31]. This study was approved by the University of British Columbia behavioral research ethics board. All use of data was approved through a Population Data BC data access request [32].

### Defining population segments

Segments were developed based on literature [6, 10, 18] and input of stakeholders including patients, decision-makers, and clinicians [33]. Two years of administrative data (fiscal year 2013/14 and 2014/15) were used to create four population segments using a combination of variables: chronic conditions, medical events suggesting medical complexity (e.g. dialysis would be an indicator of complexity among those with a diagnosis of chronic kidney disease), and markers of frailty (Table 1, supplementary file 2). For additional information about the principles and variables used to develop segments, see supplementary file 1 and file 2.

**Table 1:** Description of four population segments

Name of segment	Description
1. Low need	≤1 chronic condition and no event indicating medical complexity
2. Multiple morbidities	≥2 chronic conditions and no event indicating medical complexity
3. Medically complex	≥1 chronic condition and an event indicating complexity that is associated with a chronic condition
4. Frail	Aged ≥65, receiving frailty-based care, being deemed palliative, and/or meeting at least two criteria from the Edmonton frailty scale [34].

#### Socio-economic status (SES)

Postal codes were converted to quintiles of neighbourhood income adjusted for household size using a conversion file developed and provided by Statistics Canada [35]. Quintiles were ranked from 1 (lowest) to 5 (highest), and then dichotomized into high [3–5] and low [1, 2] SES. Based on previous work [36], neighbourhood income is considered a proxy for SES and for increased vulnerability for poor health (e.g. death) and healthcare outcomes (e.g. more hospitalizations).

### Statistical analyses

We report demographics by population segment and compare healthcare use (physician visits, hospital admissions, and medications) and associated costs in 2015–16 (the year after the population segments were classified).

#### Distribution of population segments at the practice level

To examine the distribution of population segments at the level of family physicians, patients were assigned to the primary care physician with whom they had the highest number of ambulatory visits over 3 years (2013/14 to 2015/16). In the case of a tie, patients were assigned to the provider with the higher ambulatory billings, and if still tied, to the provider most recently seen. This approach is similar to that used in other studies examining primary care in BC using administrative data [37]. Additionally, we performed an analysis of primary care physician billings by segment; this analysis included billings for all patients seen by a given family physician (not only patients that were assigned to a physician panel given that some FPs had 0 paneled patients).

#### Health care use and costs

Health care use and costs were examined by segment in the 2015–16 year. The main outcome of interest was total cost of care, which includes fee-for-service costs for family physician (FP) care, inpatient hospital care, emergency department (ED) visits, prescription medicine costs, fee-for-service costs for medical and surgical specialist care, and day surgeries. We also present information on health care use associated with costs including the number of FP visits, number of hospital inpatient separations, number of emergency department visits, and number of filled classes of medications (measured at the Anatomical Therapeutic Chemical (ATC) 4th level chemical/therapeutic/pharmacological subgroup).

#### Selected measures of attributes of primary care

We selected three exemplar measures of primary care effectiveness, or performance, based Starfield et al.’s definition [38] and previous primary care research in the BC setting [37]. Access to out of hours care and coordination of care were derived using data from 2015/16; continuity of care was derived using 3 years of data (2013/14 to 2015/16).

#### Access to out of hours FP care

The percentage of patients with FP billings for visits outside regular office hours, relying on physicians billing for out of hours care.

#### Continuity of FP care

We used the usual provider care (UPC) that characterizes the share of total physician visits to a patient’s usual FP provider. The UPC index divides the number of ambulatory visits made to the FP who provided the most visits by the total number of ambulatory FP visits for each patient and ranges from 0 to 1.0 with a higher score indicating higher continuity.

#### Coordination of FP care

We measured coordination as the percentage of total patients who saw fewer than five FPs in a given year in the ambulatory care setting.

#### Predicting health care costs

We employed a two-part Generalized Linear Model stratified by population segment to assess the relationship between several variables [age group, sex, number of chronic conditions (capped at 5; continuous variable), UPC index (continuous variable), and SES (high or low), and 2015/16 costs. Total costs were truncated at the 99th percentile within age and sex groupings to prevent outliers from overly influencing the analysis. Part one of the model predicted which factors were associated with having any health care costs in the 2015/16 year using a logit link and binomial distribution (odds ratios). Part two predicted total costs among those who had >$0 costs in the 2015/16 year, using a log link and gamma distribution (cost ratios). Both models included the following variables: age, sex, number of chronic conditions, and SES. In addition, part two of the model include three attributes of primary care: access, continuity, and coordination. We stratified the analyses by population segment because descriptive analysis suggested different relationships between SES and total costs across segments. All statistical analysis was performed using SAS software version 9.4.

## Results

A total of 3,441,393 people met our eligibility criteria and were included in subsequent analyses. The majority of the population (82%) were in the low need population segment (segment 1), while the frail segment (segment 4) was the smallest (2%) (Table 2). Just over 50% of each segment were female with the exception of segment 4, where 63% were female. The proportion of each segment > 75 years increased from 5% in segment 1 to 80% in segment 4 (Table 2). The proportion of people in the low SES group rose steadily from 40% in segment 1 to 47% in segment 4. Note that the sample sizes are reported in each table as they are not uniform across all analyses; please see table footnotes for additional information.

**Table 2:** Characteristics of the population by segment, 2015/16

	Segment 1 (82%) Low need			Segment 2 (13%) Multiple morbidities			Segment 3 (3%) Medically complex			Segment 4 (2%) Frail		
	Overall	High SES	Low SES	Overall	High SES	Low SES	Overall	High SES	Low SES	Overall	High SES	Low SES
# of people	2,807,725	1,697,453 (60.5%)	1,110,272 (39.5%)	450,197	259,060 (57.5%)	191,137 (42.5%)	117,636	62,944 (53.5%)	54,692 (46.5%)	65,835	35,082 (53.3%)	30,753 (46.7%)
Sex (%)												
Female	51.2	51.3	50.9	51.1	49.7	53.0	52.8	52.8	52.8	62.7	61.8	63.8
Age (%)												
18–44 years	45.9	44.3	48.4	6.0	5.6	6.5	25.5	25.2	25.7	.	.	.
45–64 years	39.0	40.3	37.0	35.5	35.2	35.8	39.0	38.2	39.9	.	.	.
65–74 years	10.0	10.6	9.2	29.2	30.2	27.8	17.5	18.4	16.5	19.9	20.0	19.8
75+ years	5.1	4.8	5.4	29.4	29.0	29.9	18.0	18.1	17.8	80.1	80.0	80.2
# of Chronic Conditions (Mean, SD)	0.27 (0.44)	0.27 (0.45)	0.27 (0.44)	2.50 (0.79)	2.48 (0.78)	2.53 (0.81)	2.46 (1.47)	2.42 (1.45)	2.50 (1.50)	2.79 (1.68)	2.75 (1.66)	2.85 (1.70)

All *p* ≤ 0.0001; *SES* socioeconomic status, *low SES* income quintiles 1, 2, *high SES* income quintiles 3, 4, 5

### Population segments at the practice level

**Fig. 1:**
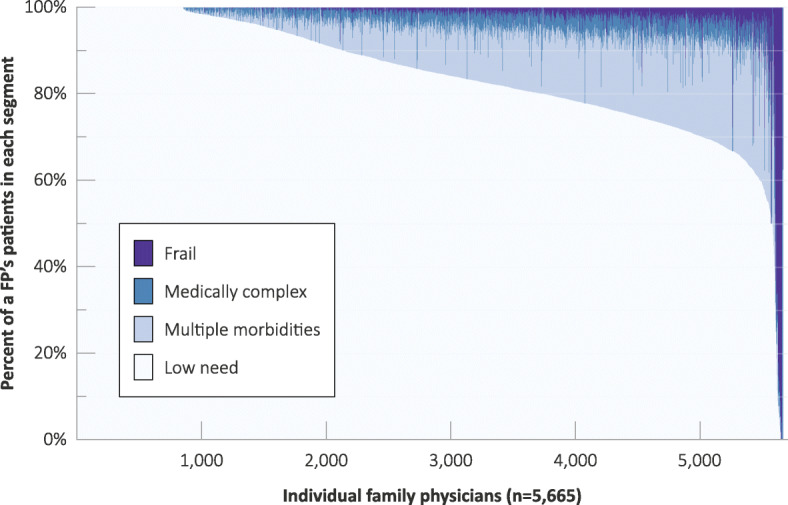
Family physician patient panels, by segment, 2015/16

**Fig. 2:**
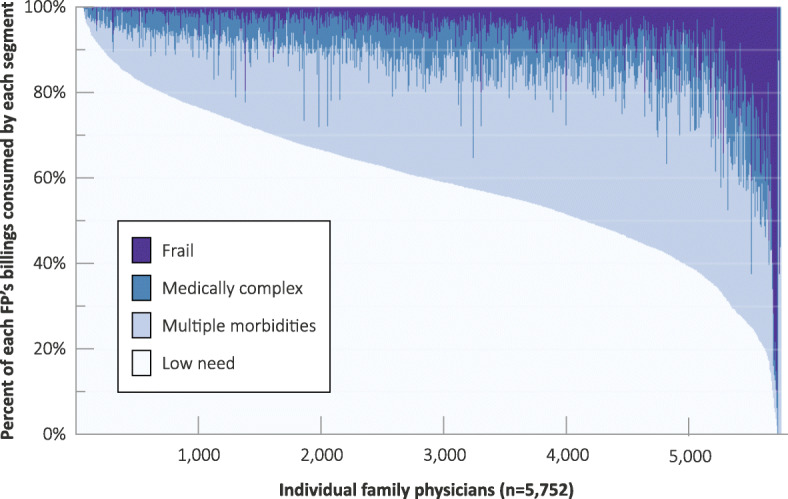
Family physician billings, by segment, 2015/16

Most primary care physicians had patient distributions across population segments that mirrored the overall picture. Others had different mixes of population segments, ranging from those that are virtually all in the healthy segment to a small number that are focused exclusively on complex and/or frail patients (Fig. 1). When we examined physician billings by segment (Fig. 2) the pattern was somewhat different in that while just under 1000 physicians had patient panels consisting of 100% low need patients, there were very few physicians with 100% costs from low need patients. Comparison of Figs. 1 and 2 demonstrates that physician billings are not proportionate to the breakdown of patient panels by segment. For example, medically complex patients or patients with multiple morbidities account for a disproportionate amount of billings relative to the percentage for these same groups in the physician panels distributions (Fig. 1).

### Variation in health services use and costs by segment

Overall health care costs included hospital costs, ED visits, day surgery, physician visits, and prescription medicines outside the hospital setting. Mean costs per person ranged from $1460 in the low need segment to $10,798 in the frail segment (Table 3). Our results suggest that population segmentation creates clear and distinct patient groups in terms of overall healthcare costs.

**Table 3:** Mean costs and use by health service type, population segment, and SES, 2015/16^a^

	Segment 1 Low need (*n* = 2,807,725)			Segment 2 Multiple morbidities (*n* = 450,197)			Segment 3 Medically complex (*n* = 117,636)			Segment 4 Frail (*n* = 65,835)		
	Overall	High SES	Low SES	Overall	High SES	Low SES	Overall	High SES	Low SES	Overall	High SES	Low SES
Mean Costs ($)												
Total FP Care (any location)	185	181	191	602	580	632	657	627	692	907	889	927
Cost of Specialist Care	222	224	219	742	750	731	1431	1401	1465	828	846	808
Inpatient Hospital Care	421	389	470	2203	2075	2378	5130	4625	5712	6741	6505	7011
Day surgeries	117	123	108	350	361	335	364	373	354	204	218	189
ED visit (estimated facility cost)	103	95	116	223	207	245	433	380	495	352	334	372
Prescription Medicines (PharmaCare + Private paid)	411	405	421	1702	1663	1755	2766	2605	2951	1766	1731	1805
Total Costs^a^	1460	1418	1525	5822	5636	6075	10,782	10,010	11,670	10,798	10,523	11,112
Use (#)												
FP visits (any location)	4.5	4.4	4.7	11.7	11.3	12.3	13.9	13.2	14.7	19.6	19.3	20.1
Hospital separations per 100 population	5.4	5.1	5.9	19.7	18.7	20.9	41.5	37.2	46.5	47.7	45.9	49.7
ED Visits per 100 population	35.3	32.6	39.4	76.2	70.7	83.7	147.9	129.5	168.9	120.0	114.0	126.9
Filled classes of medication	2.4	2.4	2.4	7.3	7.1	7.6	8.5	8.2	8.8	9.1	8.9	9.3
Selected attributes of primary care measures												
Access												
Access outside office hours: % patients with FP billing outside office hours	2.6	2.5	2.7	4.2	4.0	4.4	6.0	5.8	6.3	9.3	9.0	9.7
Continuity of Care												
UPC Index (Mean, range 0–1)	0.7	0.7	0.7	0.8	0.8	0.8	0.7	0.7	0.7	0.8	0.8	0.8
Coordination												
% patients seeing < 5 FP physicians	95.2	95.4	95.0	91.8	92.2	91.3	88.1	88.6	87.6	93.2	93.5	93.0

*All p < 0.05, ED* emergency department, *FP* family physician, *SES* socioeconomic status (*low SES* income quintiles 1, 2, *high SES* income quintiles 3, 4, 5.); *UPC* usual provider of care

^a^Total costs includes: Total FP Care, inpatient hospital care, prescription medicines, plus medical & surgical specialist care, day surgeries and ED visits

Costs for the medically complex segment (segment 3) were nearly double those of the segment with multiple morbidities (segment 2). The higher costs were driven by segment 3’s higher specialist, hospital, and medication costs relative to segment 2; costs for FP visits were similar in both segments. The medically complex and frail segments had similar overall costs but patterns of care were different with the frail segment having comparatively higher costs for hospital services and lower costs for specialist physicians and medications.

Costs were slightly higher (~ 5–7%) in the lower SES group across all segments but the difference was largest in the medically complex group (17% higher in the low SES group). In the medically complex low SES group, hospital costs were the main drivers of increased expense.

### Attributes of care by segment

The percentage of patients accessing FPs outside of regular office hours ranged from 2.6% of patients in the low need segment to 9.3% in the frail segment (Table 3). Continuity of care, as measured by UPC, was fairly stable across all segments despite the highest volume of FP use in the frail segment; however, arguably this measure may mean different things for different segments (Table 3). For example, a continuity score for the frail segment (that had the highest volume of FP use) may mean something different than the same continuity score for segments with lower volumes of FP care. There were subtle differences in coordination of care, measured as the percentage of patients seeing fewer than 5 FPs. This percentage was highest in the low need segment (95.2%) and lowest in the medically complex segment (88.1%). There were minimal differences in attributes of primary care by SES.

### Prediction of overall costs

**Fig. 3:**
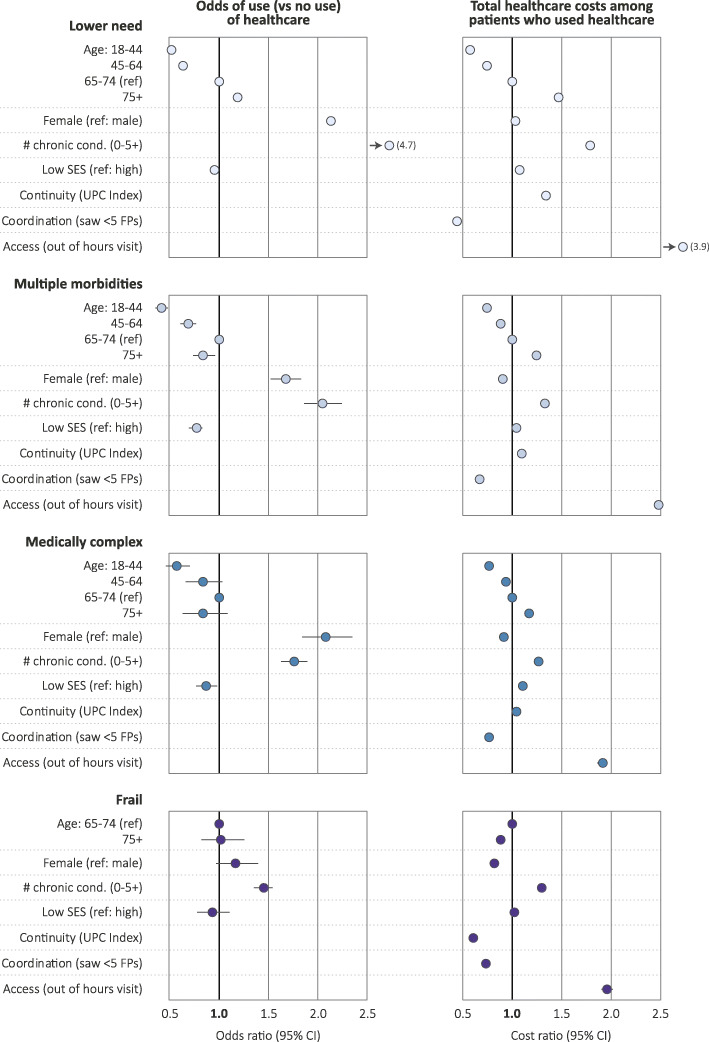
Logistic regression results, stratified by segment, 2015/16

The regression analyses demonstrated that for all segments, increasing age is associated with an increased likelihood of incurring health care costs (Table 4, Fig. 3) and with higher costs among health care users (Table 5, Fig. 3). Females have an increased likelihood of incurring any health care costs, but among those with costs, females have lower costs across all but the low need segment.

**Table 4:** Logistic regression of use (vs. no use^a^) of healthcare in BC residents, 2015/16^b^

Odds Ratio (OR) (LCL – UCL)				
	Segment 1 Low need (n = 2,558,276)	Segment 2 Multiple morbidities (*n* = 449,925)	Segment 3 Medically complex (*n* = 116,821)	Segment 4 Frail (n = 65,661)
Age (years)				
18–44	0.52 (0.51–0.53)	0.42 (0.36–0.49)	0.57 (0.46–0.71)	n/a
45–64	0.63 (0.61–0.64)	0.69 (0.61–0.77)	0.83* (0.67–1.04)	n/a
65–74	ref	ref	ref	ref
75+	1.19 (1.15–1.23)	0.84 (0.74–0.96)	0.83* (0.63–1.09)	1.02* (0.82–1.26)
Sex				
Female	2.13 (2.11–2.15)	1.67 (1.53–1.83)	2.08 (1.84–2.35)	1.16* (0.97–1.40)
Male	ref	ref	ref	ref
Number of chronic conditions (0–5+): continuous variable^c^	4.67 (4.60–4.74)	2.05 (1.87–2.25)	1.76 (1.63–1.90)	1.45 (1.36–1.55)
SES				
Low	0.95 (0.94–0.96)	0.77 (0.70–0.84)	0.87 (0.77–0.98)	0.93* (0.78–1.11)
High	ref	ref	ref	ref

*p ≥ 0.05, all other *p* < 0.05*, LCL* lower confidence limit*, UCL* upper confidence limit; *SES socioeconomic status* (*low SES* income quintiles 1, 2, *high SES* income quintiles 3, 4, 5.)

^a^No health care use is defined as $0 in health care costs in 2015/16.The number of individuals with $0 in health care costs in 2015/16 varies by segment: Segment 1 = 264,375; Segment 2 = 2035; Segment 3 = 1095; Segment 4 = 505

^b^Note that this table excludes individuals with no FP visits in the 3 years of data used to create the continuity of care measure (UPC). The number of people excluded varies by segment: Segment 1 = 249,449; Segment 2 = 272; Segment 3 = 815; Segment 4 = 174

^c^Number of chronic conditions was treated as a continuous variable given that the number of chronic conditions varies by segment (e.g., by definition, segment 1 has fewer chronic conditions than segment 4); please see Supplementary File 3 (Table 1a and b) for analyses where chronic conditions were treated as categorical variables; we note that this did not change our findings

**Table 5:** Total healthcare costs among patients who used the BC healthcare system, 2015/16^a^

Cost Ratio (CR) (LCL – UCL)				
	Segment 1 Low need (n = 2,293,901)	Segment 2 Multiple morbidities(*n* = 447,890)	Segment 3 Medically complex (*n* = 115,726)	Segment 4 Frail (n = 65,156)
Age (years)				
18–44	0.57 (0.56–0.57)	0.75 (0.74–0.76)	0.77 (0.75–0.78)	n/a
45–64	0.74 (0.74–0.75)	0.88 (0.87–0.88)	0.94 (0.92–0.96)	n/a
65–74	ref	ref	ref	ref
75+	1.47 (1.45–1.48)	1.24 (1.23–1.25)	1.17 (1.15–1.19)	0.88 (0.86–0.90)
Sex				
Female	1.03 (1.03–1.03)	0.90 (0.90–0.91)	0.91 (0.90–0.93)	0.82 (0.81–0.84)
Male	ref	ref	ref	ref
Number of chronic conditions (0–5+): continuous variable^b^	1.79 (1.79–1.80)	1.33 (1.32–1.33)	1.27 (1.26–1.28)	1.30 (1.30–1.31)
SES				
Low	1.07 (1.06–1.07)	1.04 (1.03–1.05)	1.11 (1.09–1.12)	1.02 (1.00–1.04)
High	ref	ref	ref	ref
Continuity index (UPC)	1.34 (1.33–1.35)	1.09 (1.07–1.10)	1.04 (1.01–1.07)	0.61 (0.58–0.64)
Coordination: number of FPs				
Saw < 5 FPs	0.45 (0.45–0.46)	0.67 (0.66–0.68)	0.77 (0.75–0.78)	0.73 (0.70–0.76)
Saw > = 5 FPs	ref	ref	ref	ref
Access: out-of-hours FPs				
Yes	3.91 (3.87–3.94)	2.48 (2.44–2.52)	1.91 (1.86–1.96)	1.96 (1.90–2.02)
No	ref	ref	ref	ref

*p ≥ 0.05, all other *p* < 0.05; *LCL* lower confidence limit, *UCL* upper confidence limit, *FP* family physician, *SES* socioeconomic status, *UPC* usual provider of care

^a^As in Table 4a, this table excludes individuals with no FP visits in the 3 years of data used to create the continuity of care measure (UPC). This table also excludes individuals with $0 costs in 2015/16 which varies by segment: Segment 1 = 264,375; Segment 2 = 2035; Segment 3 = 1095; Segment 4 = 505

^b^Number of chronic conditions was treated as a continuous variable given that the number of chronic conditions varies by segment (e.g., by definition segment 1 has fewer chronic conditions than segment 4); please see Supplementary File 3 (Table 1a and b) for analyses where chronic conditions were treated as categorical variables; we note that this did not change our findings

Across all segments, those in lower SES quintiles were less likely to use health care services (Table 4, Fig. 3) but had higher costs among the users (Table 5, Fig. 3). The exception was the frail group, which showed limited variability across SES.

The regression analyses demonstrate that those with a higher number of chronic conditions were more likely to use health care services (Table 4, Fig. 3) and had higher costs among the users (Table 5, Fig. 3). To test for the linearity of this effect, we ran a logistic regression model with number of chronic conditions as categorical (Supplementary File 3, Table 1a and b). This analysis did not substantively change our findings except for showing that those in segment 3 and 4 with a smaller number of chronic conditions (0–1) were less likely to use health care services and have lower costs among the users. These additional analyses also showed that the addition of one chronic condition had different implications in terms of health care use and costs for different segments. For example, an increase from zero to one chronic condition in segment 1 was associated with an increased likelihood of health care use and costs among the users and the magnitude of this effect seemed to be larger than an increase of one chronic condition (for example, an increase from 3 to 4 or 4 to 5 chronic conditions) in the more complex segments [2 through 4]. For segments 2 through 4, the association between number of chronic conditions and health care use and costs was relatively linear.

In terms of the attributes of primary care, continuity of care was associated with lower costs for frail population segment only (cost ratio = 0.61). Out of regular office hours FP visits were associated with higher health care costs across all segments, and the magnitude of this effect was largest in the low need segment (cost ratio = 3.91). Finally, coordination of care (seeing fewer than 5 FPs in a given year) was associated with lower costs across all segments and the magnitude of this effect was greatest for the low need segment. In other words, disorganized care (seeing 5 or more FPs) is associated with higher costs.

## Discussion

Four mutually exclusive and exhaustive population segments designed to capture need for primary health care services are distributed differently across physician practices, suggesting that these segments may help understand variations in practice-level costs and patterns of care. These population segments showed expected variation in terms of use/costs of health care services while differences in measures of attributes of primary care were not as pronounced as expected. Consistent with previous studies, we found that a small proportion of the population accounts for the largest proportion of overall health care costs [8]. As patient complexity increases, variation within population segments of health care costs also increase with the medically complex and frail segments having the greatest variation in health care costs. Our proxy measure for SES shows that lower income is associated with lower likelihood of access to health care, but higher use among those who have any use; this is largely consistent with existing Canadian [39] and international [40] literature. Our finding that low SES was associated with higher costs across all segments but more pronounced in the medically complex segment is consistent with other research that suggests that SES plays a role in managing changes in health status and leads to health inequities [41, 42].

Age and number of chronic conditions were associated with health care costs but the patterns were different by segment suggesting that population segments provide nuanced information about health care use/costs [43]. This also suggests that age or number of chronic conditions does not always predict increased health care costs and that segments could be a useful value-add for better addressing otherwise unmeasured constructs that affect health and healthcare use. Given that segments were defined using 2 years of data and health care costs/primary care attributes were examined in the subsequent year, segments may be a useful tool to anticipate health system needs and to inform system planning. Using segments for this purpose means that interventions can be aimed at service needs for particular groups rather than targeting interventions based on single medical conditions.

Variations in the attributes of primary care across segments further underline the potential utility of disaggregated reporting. For example, the relationship between out-of-office care and higher costs in the healthy and frail segments might point to different underlying issues; for healthier individuals this may reflect a need for better coordination of services and/or structure of office hours, while for frail individuals, out-of-office care may be a necessary component of care for their complex needs. However, we note that future research should test the utility of the segments for other important attributes of primary care such as effectiveness, patient-centeredness, and comprehensiveness [38, 44].

Health system planners could use information on population segments at the community or regional level to provide and tailor supports to primary care clinicians and regions. For example, primary care population segments provide an opportunity for resourcing collaborative interdisciplinary healthcare teams and integrated team pathways, particularly for practices with a disproportionate percentage of complex and/or frail patients [45]. Integrative approaches and sharing of responsibility and accountability could address some of the unique challenges within the different segments [46].

Our analyses relied on administrative data and are subject to the usual limits of data that are not collected specifically for research purposes such as a lack of clinical information and time lags in data access. Generally, administrative data are retrospective and if such an approach is to be used to influence decision making, it will be important to move towards real-time analyses and effectively track the highest need, most vulnerable populations [47]. Administrative data are population-based but mainly capture fee-for-service primary care services. It would be useful to examine other models of primary care such as capitation [48] using population segments given the expected differences in need for service across segments. We constructed four population segments and there are of course many other options for defining specific segments of interest and further research should address the robustness of these findings when applied to different population segment definitions in other jurisdictions [6–8]. Our approach would be strengthened by linking administrative data with other data sources to capture elements of performance such as patient-reported outcome and experience measures to more accurately capture patient needs and experiences, examine factors such as the presence of carers and social supports, patient behaviours and traits that may be more predictive of health care needs than medical complications [19]. Having access to these data sets would enable us to enhance our definition of vulnerability, as our SES measure (based on postal code) only scratched the surface of vulnerability [17].

## Conclusion

In conclusion, these four distinct population segments have potential utility for primary care performance measurement and reporting. Our approach could be used to develop and tailor information on primary care performance for different groups such as health care providers and decision makers such that segments could be used for practice management and quality improvement efforts. This information also provides a useful springboard for further in-depth analyses that help elucidate the underlying causes of variations in care.

## Supplementary information

**Additional file 1:Supplementary File 1.**

**Additional file 2: Supplementary File 2.** Definitions for chronic conditions and complications to derive segments 2, 3 and 4.

**Additional file 3: Supplementary File 3.** Regression analysis model with chronic conditions as a categorical variable.

## Data Availability

The data that support the findings of this study are available from Population Data BC (https://www.popdata.bc.ca/) but restrictions apply to the availability of these data, which were used for the current study under Data Stewards’ approval and Research Agreements with Data Stewards, and so are not publicly available. Data are however available from Population Data BC upon reasonable request and with permission of the Data Stewards.

## Abbreviations

ATC: Anatomical therapeutic chemical
BC: British Columbia
CHF: Congestive heart failure
CR: Cost Ratio
DAD: Discharge abstracts database
ED: Emergency department
FP: Family physician
LHA: Local health area
MSP: Medical services plan
NACRS: National Ambulatory Care Reporting System
PREMS: Patient reported experience measures
PROMS: Patient reported outcome measures
PopData BC: Population Data BC
SES: Socioeconomic status
UPC: Usual provider care
