# Supplementary material for: Population segments as a tool for health care performance reporting: an exploratory study in the Canadian province of British Columbia
Source: BMC Fam Pract. 2020 May 31;21:98. doi: 10.1186/s12875-020-01141-w (PMC7262753; doi:10.1186/s12875-020-01141-w)
Supplement: Supplementary file 1 — Additional file 1:Supplementary File 1. [file 12875_2020_1141_MOESM1_ESM.docx]

**Supplementary File 1**

*Principles for defining the population segments.* Four population segments were developed based on relevant literature^6,10,18^ and input of stakeholders including patients, decision-makers, and clinicians. ^34^ The segments were developed to:

1. Capture the majority of people who receive primary care services and assign them to mutually exclusive segments;
2. Predict primary care utilisation in subsequent years;
3. Create groups with similar needs for primary care and distinguish between lower need patients’ whose care is fully provided by primary care and more complex patients whose care is coordinated by primary care but involves other parts of the health care system. Our goal was segmentation according to need, both for care and for coordination;
4. Enable comparisons at different levels of aggregation (e.g., practice-level and region-level reporting) to ensure that performance information by segments could be used to support multiple stakeholders involved in care delivery.^50^

*Health care variables used to create the population segments.* Segments were defined using a combination of chronic conditions, medical events suggesting medical complexity, and markers of frailty. The four segments used here are:

1. Low need: ≤1 chronic conditions and no event indicating medical complexity;
2. Multiple morbidities: ≥2 chronic conditions and no event indicating medical complexity. We focused on the following 15 chronic conditions and used administrative data definitions based on similar studies:^51^ arthritis, asthma, cancer, cerebrovascular disease, chronic kidney disease, chronic liver disease, chronic neurogenerative diseases, chronic obstructive pulmonary disease, congestive heart failure, depression, diabetes, hypertension, ischemic heart disease and osteoporosis;
3. Medically complex: ≥1 chronic condition and an event indicating complexity that is associated with a chronic condition. Medical complexity definitions were developed for each chronic condition based on clinical input and relevant literature.^51^ Computational techniques to determine the most frequently occurring combinations and permutations of chronic diseases were considered however, recent research suggests that there are far too many combinations to identify a common cluster of chronic conditions that would account for the majority of the population^51^.
4. Frail: derived based on ongoing work in a separate project^52,49^ and included being aged 65 or older, receiving facility-based care, being deemed palliative, and/or meeting at least two criteria from the Edmonton frailty scale.^49^
