# Supplementary material for: Population segments as a tool for health care performance reporting: an exploratory study in the Canadian province of British Columbia
Source: BMC Fam Pract. 2020 May 31;21:98. doi: 10.1186/s12875-020-01141-w (PMC7262753; doi:10.1186/s12875-020-01141-w)
Supplement: Supplementary file 2 — Additional file 2: Supplementary File 2. Definitions for chronic conditions and complications to derive segments 2, 3 and 4. [file 12875_2020_1141_MOESM2_ESM.docx]

**Supplementary File 2. Definitions for chronic conditions and complications to derive segments 2, 3 and 4**

**Table 1.** Identifying medical complications using British Columbia administrative data

|  |  | **CLASSIFCATION OF COMPLICATIONS** | | |
| --- | --- | --- | --- | --- |
| **Chronic Condition** | **Events that may indicate complications related to chronic condition** | **Visits and Procedures: Physician Claims** [Medical Services Plan Payment Information](https://www.popdata.bc.ca/data/health/msp) File | **Hospital Admissions ICD-10-CA: Discharge Abstract Database** | **Medicines:**  **Pharmanet** |
| Arthritis (including osteoarthritis) | Count as chronic condition only, no complexity definition as arthritis does not usually have complications. | | | |
| Asthma | ≥1 hospital admission (asthma most responsible for admission)  OR  ≥2 visits with respirologist within a 12-month period | Multiple visits with respirologist: Practitioner specialty = 49 | -Asthma (J45)  -Status asthmaticus (J46). |  |
| Cancer | Count as chronic condition only. Cancer will contribute to counts of chronic diseases for morbidity burden; however active cancer treatment not usually delivered in primary care. | | | |
| Cerebrovascular disease | ≥1 hospital admission for stroke (most responsible or primary reason for admission)  OR  ≥1 hospital admission (cerebrovascular disease most responsible for admission) | N/A | Stroke:  -Subarachnoid hemorrhage (I60)  -Intracerebral hemorrhage (I61)  -Other non-traumatic intracranial hemorrhage (I62)  -Cerebral infarction (I63)  -Stroke, not specified as hemorrhage or infarction (I64).  -Central retinal artery occlusion (H34.1)  Cerebrovascular disease:  -Vertebro-basilar artery syndrome (G45.0)  -Carotid artery syndrome (G45.1)  - Multiple and bilateral precerebral artery syndromes (G45.2)  - Amaurosis fugax (G45.3)  - Other transient cerebral ischaemic attacks and related syndromes (G45.8)  - Transient cerebral ischaemic attack, unspecified (G45.9) |  |
| Chronic kidney disease (renal failure) | Dialysis  OR  Kidney transplant | Dialysis fees for acute renal failure, chronic renal failure (haemodialysis and peritoneal dialysis) and home dialysis: 33750, 33751, 33752, 33708, 33756, 33758, 77380, 33723, 33759, 33761) | CCI procedure codes  Kidney transplant (1.PC.85 and 1.0K.85)  OR  Dialysis codes 1.PZ.21 (Dialysis, urinary system including kidneys) |  |
| Chronic liver disease (hepatic failure)* | Complications (most responsible or primary reason for admission): hepatic encephalopathy  OR  bleeds (GI, oesophageal varices, any bleeding complication)  OR  renal events (hepatorenal syndrome)  OR  spontaneous bacterial peritonitis  OR  Ascites  OR  Portal Hypertension  OR  Liver transplant | N/A | -Hepatic encephalopathy (K72)  -Oesophageal varices (I85)  -Renal failure (N17, N18, N19, Z49.2, Z99.2)  -GI bleeds(I85.0, K92.2);  -Spontaneous bacterial peritonitis (K65.2)  -Hepatorenal syndrome (K76.7)  -Ascites (R18)  -Portal hypertension (K76.6)  -liver transplant (CCI 1.0A.85) |  |
| Chronic Neurodegenerative Diseases (Multiple Sclerosis, Amyotrophic Lateral Sclerosis, Parkinson’s disease, Alzheimer’s disease, stroke or other brain injury with a permanent neurological deficit, paraplegia or quadriplegia etc.)* | In aged care/long-term care facility | Fee item:  - Visit nursing home one or multiple patients (00114)  - Nursing home visit – 1 patient when specially called (00115)  - LTC facility visit – first visit of the day bonus, extra (13334) | Separation Disposition = 02 transferred to a LTC facility  BC Level From or BC Level To = I Intermediate / Personal Care | Pharmacare Plan B (for permanent residents of licensed residential care facilities, as long as the facility has asked PharmaCare to be on the list of Plan B facilities). |
| Chronic obstructive pulmonary disease | ≥1 hospital admission (COPD most responsible);  OR  ≥2 visits with respirologist within a 12-month period | Multiple visits with respirologist: Practitioner specialty = 49 | -Simple and mucopurulent chronic bronchitis (J41)  -Unspecified chronic bronchitis (J42)  -Emphysema (J43)  -Other chronic obstructive pulmonary disease (J44) |  |
| Congestive heart failure | multiple hospital admissions (>=2 within a 12-month period) (CHF most responsible or primary) | N/A | -Heart failure (I50). |  |
| Depression | ≥1 hospital psychiatry (depression most responsible)  OR  ≥2 outpatient psychiatry within a 12-month period | multiple outpatient psychiatry: Practitioner specialty = 03 | -Depressive episode (F32)  -Recurrent depressive disorder (F33)  -Mixed anxiety and depressive disorder (F41.2) |  |
| Diabetes mellitus | sequelae: stoke, MI, amputation (limbs), renal failure [as most responsible for admission or primary]  OR  other diabetes complications (renal, peripheral, ophthalmology)[ as most responsible for admission or primary]  OR  ≥1 hospitalization with diabetes most responsible | N/A | -stroke (I63)  -MI (I21, I22)  -amputation (Z89.x)  -renal failure (N17, N18, N19, Z49.2, Z99.2)  OR diabetes complications (E10-E14 with 4^th^ digit equal to: renal 0.2 , opth 0.3, peripheral 0.5, multiple 0.7) |  |
| Hypertension | sequelae:  >=1 hospitalization (most responsible or primary) for  stroke  OR  Aneurysm  OR  heart failure  OR  MI  Or  ≥1 hospitalization (with hypertension most responsible) | N/A | Sequelae (most responsible or primary):  -Subarachnoid hemorrhage (I60)  -Intracerebral hemorrhage (I61)  -Other non-traumatic intracranial hemorrhage (I62)  -Cerebral infarction (I63)  -Stroke, not specified as hemorrhage or infarction (I64)  -Acute myocardial infarction (I21)  -Subsequent myocardial infarction (I22)  -Cerebral aneurysm, non-ruptured (I67.1)  -Aneurysm of heart (I25.3)  -Coronary artery aneurysm and dissection (I25.4)  -Heart failure (I50).  Hypertension (most responsible):  -Essential (primary) hypertension (I10)  -Hypertensive heart disease (I11)  -Hypertensive renal disease (I12)  -Hypertensive heart and renal disease (I13)  -Secondary hypertension (I15) |  |
| Inflammatory bowel disease | ≥1 hospitalization (with inflammatory bowel disease most responsible) |  | -Crohn disease [regional enteritis] (K50)  -Ulcerative colitis (K51). |  |
| Ischemic heart disease | Count as chronic condition only. Hard to define "more complex" ischemic heart disease. Heart attacks may indicate complexity but are part of the diagnosis definition already and the number of heart attacks is not necessarily correlated with complexity; often hard to evaluate the severity of heart attacks with available data. Arrhythmia or heart failure may be possibilities but again, hard to define and/or isolate as a complication associated with ischemic heart disease. | | | |
| Osteoporosis | Fractures (most responsible or primary reason for hospital admission) |  | - Osteoporosis with current pathological fracture (M80) -Fracture of skull and facial bones (S02) -Fracture of neck (S12)  -Fracture of rib(s), sternum and thoracic spine (S22),  -Fracture of lumbar spine and pelvis (S32)  -Fracture of shoulder and upper arm (S42)  -Fracture of forearm (S52)  -Fracture at wrist and hand level (S62)  -Fracture of femur (S72)  -Fracture of lower leg, including ankle (S82)  -Fracture of foot, except ankle (S92)  -Fractures involving multiple body regions (T02)  -Fracture of upper limb, level unspecified (T10). |  |

**Table 2.** ICD-9 and ICD-10 codes for chronic conditions.

| **Chronic Condition** | **ICD9**  **Physician Claims: Medical Services Plan Payment Information File** | **ICD-10-CA**  **Hospital Discharge Abstract Database**** |
| --- | --- | --- |
| Arthritis (including osteoarthritis) | 715, 727, 729, 710, 720, 274, 716, 711, 718, 728, 739 | M00-M03, M07, M10, M11-M14, M15-M19, M20-M25, M30-M36, M65-M79 |
| Asthma | 493  BC-specific Complex Care Diagnostic codes: A585, A491, A430, A428, A414, A250 | J45, J46 |
| Cancer | 140-172, 174-208 | C00-C26, C30-C43, C45-C97 |
| Cerebrovascular disease | 362.3, 430, 431, 433.x1, 434, 435, 436  BC-specific Complex Care Diagnostic codes: C585, C573, C491, A430, N430, R430, H430, D430, I430  (Exclude if any traumatic brain injury code found on same date or person in hospital on that date with either traumatic brain injury codes or "rehabilitation care code” (Z50) as the primary hospital discharge diagnosis) | G45.0, G45.1, G45.2, G45.3, G45.8, G45.9, H34.1, 160, I61, I63, I64  Exclude if any traumatic brain injury code is used on the same hospital record or the "rehabilitation care code” (Z50) is the primary hospital discharge diagnosis. |
| Chronic kidney disease (renal failure) | 582, 583, 584, 585, 586, 587, 589   BC-specific Complex Care Diagnostic codes: K573, A585, C585, N585, R585, H585, D585, I585, R491 | N01-N07, N18, N19, N26, N27 |
| Chronic liver disease (hepatic failure)* | 571, 573  BC-specific Complex Care Diagnostic codes: C573, K573, N573, R573, H573, D573, I573 | K70, K71, K72 |
| Chronic Neurodegenerative Diseases (Multiple Sclerosis, Amyotrophic Lateral Sclerosis, Parkinson’s disease, Alzheimer’s disease, stroke or other brain injury with a permanent neurological deficit, paraplegia or quadriplegia etc.)* | 290, 330-337, 340-344, 800-804, 850-854  BC-specific Complex Care Diagnostic codes: N430, N585, N573, N519, N428, N250, N414 | F00, F01, F02, F03, G11, G12, G20-G26, G30-G32, G35, G80-G83, S02.0, S02.1, S02.2, S02.3, S02.4, S02.6, S02.8, S02.9, S06 |
| Chronic obstructive pulmonary disease | 491, 492, 496  BC-specific Complex Care Diagnostic codes:  A491, C491, H491, D491, I491, R491 | J41-J44 |
| Congestive heart failure | 428   BC-specific Complex Care Diagnostic codes: H430, H585, H573, H491, H250, A428, N428, R428, I428  To quality, the 2 (or more) diagnoses were required to be within a 1-year period. | I50 |
| Depression | 311, 296  To quality, the 2 (or more) diagnoses were required to be within a 1-year period. | F32, F33, F412 |
| Diabetes mellitus  Exclude gestational diabetes by disregarding occurrences of these codes occurring 120 days before or 180 days after any record(s) containing gestational diagnostic codes | 250  BC-specific Complex Care Diagnostic codes: D430, D491, D573, D585, A250, N250, R250, H250, I250 | E10-E14 |
| Hypertension  Exclude pregnancy-induced hypertension by disregarding occurrences of these codes occurring 120 days before or 180 days after any record(s) containing gestational diagnostic codes | 401, 402, 403, 404, 405 | I10, I11, I12, I13, I15 |
| Inflammatory bowel disease | 555, 556 | K50, K51 |
| Ischemic heart disease | 410, 411, 412, 413, 414  BC-specific Complex Care Diagnostic codes: I430, I585, I573, I428, I491, I250, A414, N414, R414  To quality, the 2 (or more) diagnoses were required to be within a 1-year period. | I20, I21, I22, I23, I24, I25 |
| Osteoporosis | 733 | M81, M82 |

**Codes and definitions for identification of frail segment (segment 4)**

Those aged 65+ meeting at least ONE of the three decision rules listed below are considered frail. Please note that decision rules one and two are ‘stand alone’ – they do not require additional information to be identified as frail. To be identified using decision rule three, a**t least two conditions are required in combination, within a rolling 2-year period**.

**IDENTIFICATION RULE #1**

/* LONG TERM CARE RESIDENT-NURSING HOME RESIDENT */ OR RECEIVING ASSISTED LIVING

To be considered frail, only one of the following is required.

| **Variable Definition** | **Field Name** | **Value** |
| --- | --- | --- |
| DISCHARGE ABSTRACTS (DAD) | | |
| Institution patient was admitted from | BCLVLFROM | I=Intermediate/ Personal Care |
| Institution where patient was discharged to | BCLVLTO | I=Intermediate/ Personal Care |
| Discharge | SEPDISP | 02= transferred to a long-term care facility |
| PHYSICIAN BILLINGS/CLAIMS | | |
| Location where the service was provided | SERVLOC | C = Residential care / assisted living residence |
| Service | FEEITEM | 00114= Visit nursing home one or multiple patients  00115= Nursing home visit – 1 patient when specially called  13334= LTC facility visit – first visit of day bonus, extra |
| PHARMACARE DATA | | |
| Pharmacare Plan for LTC residents | ACCOUNT_CD | B = Plan B covers prescription drugs for Permanent Residents of Licensed Residential Care Facilities. Both public and private residential care facilities can register for Plan B |

**IDENTIFICATION RULE #2**

/* TERMINALLY ILL */

We are looking at services provided to two cohorts of frail seniors. Those who had died and others who, from the information we have, were still living. Both groups will be handled the same way as to whether or not they were considered terminally ill during the time period and therefore frail.

| **Variable definition** | **Field name** | **Value** |
| --- | --- | --- |
| DISCHARGE ABSTRACTS (DAD) | | |
| Main patient service | PATSERV | 58 = palliative care |
| Diagnosis codes (1-3) | DIAGX1-3 | ICD-10-CA Z51.5 = Encounter for palliative care |
| CMG | CMG | 810 = Palliative Care |
| PHYSICIAN BILLINGS/CLAIMS | | |
| Diagnosis codes (1-3) | ICD9_1-3 | ICD9 V667 =Encounter for palliative care |
| Service | FEEITEM | BC specific fee items:  00127 terminal care facility visit  14063 Palliative care planning fee-general practice  96963 APB Palliative care planning fee  96163 PBF Palliative care planning fee  96969 APB Palliative care telephone/email follow-up management fee  96169 PBF Palliative care telephone/email follow-up management fee |
| PHARMACARE DATA | | |
| Pharmacare Palliative Care Plan | ACCOUNT_CD | P = Palliative Care Benefits Program (Plan P) for BC palliative care residents who wish to receive palliative care at home |

**IDENTIFICATION RULE #3 a-g**

/* INDICES FROM THE EDMONTON FRAIL SCALE (Modified for BC context) plus SERVICE UTILIZATION*/

Seniors are considered frail if they met at least TWO or more of the following domains from 3a to 3f:

3a. COGNITIVE IMPAIRMENT: Any one of the ICD9/ICD-10-CA diagnoses listed

| **Definition** | **ICD9/ICD10-CA** | |
| --- | --- | --- |
| DISCHARGE ABSTRACTS (DAD) AND/OR PHYSICIAN BILLINGS/CLAIMS DXCODES1-3 | | |
| Senile dementia, uncomplicated | ICD9  ICD-10-CA | 290  F03 |
| Dementia in Alzheimer's disease | ICD9  ICD-10-CA | 331.0  G30 |
| Vascular dementia | ICD9  ICD-10-CA | 290.4 (captured above under 290)  F01 |
| Dementia in other diseases classified elsewhere | ICD9  ICD-10-CA | 294.1  F02 |
| Unspecified dementia | ICD9  ICD-10-CA | 294.2  F03 |
| Cerebral generations usually manifest in childhood | ICD9  ICD-10-CA | 330  G94 |
| Other cerebral degenerations including Alzheimer’s | ICD9  ICD-10-CA | 331, 332, 333, 334, 335  G31, G32 |
| Senility without mention of psychosis | ICD9  ICD-10-CA | 797  R54 |
| Delirium | ICD9  ICD-10-CA | 293  F05 |

3b. INCONTINENCE: Any one of the ICD9/ICD-10-CA diagnoses listed

| **Definition** | **ICD9/ICD10-CA** | |
| --- | --- | --- |
| DISCHARGE ABSTRACTS (DAD) AND/OR PHYSICIAN BILLINGS/CLAIMS DXCODES1-3 | | |
| Urinary | ICD9  ICD-10-CA | 788.3  R32 |
| Fecal | ICD9  ICD-10-CA | 787.6  R15 |

3c. FALLS: Any one of the ICD-10-CA diagnoses listed

| **Definition** | **ICD10-CA** | |
| --- | --- | --- |
| DISCHARGE ABSTRACTS (DAD) DXCODES1-3 | | |
| Various falls  Only count if associated with hospitalization. | ICD-10-CA | W01, W05, W06-W19 |

3d. NUTRITION ISSUES: Any one of the ICD9/ICD-10-CA diagnoses listed

| **Definition** | **ICD9/ICD10-CA** | |
| --- | --- | --- |
| DISCHARGE ABSTRACTS (DAD) AND/OR PHYSICIAN BILLINGS/CLAIMS DXCODES1-3 | | |
| Abnormal weight loss; underweight; other concerns | ICD9  ICD-10-CA | 783.0, 783.2, 783.3, 783.9  R63.0, R63.3, R63.4, R63.6, R63.8 |
| Failure to thrive (adult) | ICD9  ICD-10-CA | 783.7  R62.7 |
| Cachexia | ICD9  ICD-10-CA | 799.4  R64 |

3e. FUNCTIONAL PERFORMANCE: Any one of the ICD9/ICD-10-CA diagnoses listed

| **Definition** | **ICD9/ICD10-CA** | |
| --- | --- | --- |
| DISCHARGE ABSTRACTS (DAD) AND/OR PHYSICIAN BILLINGS/CLAIMS DXCODES1-3 | | |
| Abnormality of gait | ICD9  ICD-10-CA | 781.2  R26 |
| Difficulty in walking | ICD9  ICD-10-CA | 719.7  R26.2 |
| Muscular wasting and disuse atrophy | ICD9  ICD-10-CA | 728.2  M62.50 |
| Muscular weakness | ICD9  ICD-10-CA | 728.87  M60.1 |
| Pressure ulcer | ICD9  ICD-10-CA | 707  L89, L97, L98.4 |
| Malaise and fatigue /debility | ICD9  ICD-10-CA | 780.7, 799.3  R53, G93.3 |
| Other diseases of spinal cord | ICD9  ICD-10-CA | 336  G95 |

3f. TARGETED HEALTH SERVICE UTILIZATION: Any one of the following service types

| **Definition** | **Data file** | **Fields** |
| --- | --- | --- |
| One or more geriatrician billing claim | Physician billing | Provider type  = 24 Geriatric medicine |
| One or more geriatrician service claim | DAD | Main patient service (PatServ)  = 72 geriatrics |
| One or more provider home visit (any type of physician) | Physician billings | Location of service (ServLoc)  = R Patients home |
