# Supplementary material for: Population segments as a tool for health care performance reporting: an exploratory study in the Canadian province of British Columbia
Source: BMC Fam Pract. 2020 May 31;21:98. doi: 10.1186/s12875-020-01141-w (PMC7262753; doi:10.1186/s12875-020-01141-w)
Supplement: Supplementary file 3 — Additional file 3: Supplementary File 3. Regression analysis model with chronic conditions as a categorical variable. [file 12875_2020_1141_MOESM3_ESM.docx]

**Supplementary File 3: Regression analysis model with chronic conditions as a categorical variable**

| Table 1a. Logistic regression of use (vs. no use) of healthcare in BC residents, 2015/16 | | | | |
| --- | --- | --- | --- | --- |
| Odds Ratio (OR) (LCL – UCL) | | | | |
|  | **Segment 1**  Low need  (n= 2,558,276) | **Segment 2**  Multiple morbidities  (n=449,925) | **Segment 3**  Medically complex  (n=116,821) | **Segment 4**  Frail  (n= 65,661**)** |
| Age (years) | | | | |
| 18-44 | 0.52 (0.51 - 0.53) | 0.42 (0.36 - 0.49) | 0.61 (0.48 - 0.76) | n/a |
| 45-64 | 0.63 (0.61 - 0.64) | 0.69 (0.61 - 0.77) | 0.84* (0.67 - 1.04) | n/a |
| 65-74 | ref | ref | ref | ref |
| 75+ | 1.19 (1.15 - 1.23) | 0.84 (0.74 - 0.96) | 0.86* (0.65 - 1.13) | 1.03* (0.83 - 1.27) |
| Sex | | | | |
| Female | 2.13 (2.11 - 2.15) | 1.67 (1.53 - 1.83) | 2.08 (1.84 - 2.35) | 1.16* (0.96 - 1.39) |
| Male | ref | ref | ref | ref |
|  | | | | |
| Number of chronic conditions | | | | |
| 0 | ref | n/a | n/a | n/a |
| 1 | 4.67 (4.60-4.74) | n/a | 0.39 (0.33-0.46) | n/a |
| 0-1 | n/a | n/a | n/a | 0.43 (0.34-0.54) |
| 2 | n/a | ref | ref | ref |
| 3 | n/a | 2.30 (2.02-2.62) | 1.44 (1.13-1.85) | 0.92* (0.69-1.22) |
| 4 | n/a | 4.45 (3.31-5.97) | 1.82 (1.32-2.51) | 1.38* (0.96-1.96) |
| 5+ | n/a | 3.66 (2.33-5.77) | 2.50 (1.70-3.67) | 1.81 (1.24-2.65) |
| SES (socioeconomic status) | | | | |
| Low | 0.95 (0.94 - 0.96) | 0.77 (0.70 - 0.84) | 0.87 (0.77 - 0.98) | 0.94* (0.78 - 1.12) |
| High | ref | ref | ref | ref |
| *Note: * p ≥ 0.05, all other p < 0.05* | | | | |

| Table 1b. Total healthcare costs among patients who used the BC healthcare system, 2015/16 | | | | |
| --- | --- | --- | --- | --- |
| Cost Ratio (CR) (LCL – UCL) | | | | |
|  | **Segment 1**  Low need  (n= 2,293,901) | **Segment 2**  Multiple morbidities  (n=447,890) | **Segment 3**  Medically complex  (n=115,726) | **Segment 4**  Frail  (n=65,156) |
| Age (years) | | | | |
| 18-44 | 0.57 (0.56 - 0.57) | 0.75 (0.74 - 0.76) | 0.78 (0.76 - 0.79) | n/a |
| 45-64 | 0.74 (0.74 - 0.75) | 0.88 (0.87 - 0.88) | 0.95 (0.93 - 0.96) | n/a |
| 65-74 | ref | ref | ref | ref |
| 75+ | 1.46 (1.45 - 1.48) | 1.24 (1.23 - 1.25) | 1.17 (1.15 - 1.19) | 0.88 (0.86 - 0.90) |
| Sex | | | | |
| Female | 1.03 (1.02 - 1.03) | 0.90 (0.90 - 0.91) | 0.91 (0.90 - 0.92) | 0.82 (0.80 - 0.83) |
| Male | ref | ref | ref | ref |
|  | | | | |
| Number of chronic conditions | | | | |
| 0 | ref | n/a | n/a | n/a |
| 1 | 1.80 (1.79 - 1.80) | n/a | 0.76 (0.74-0.77) | n/a |
| 0-1 | n/a | n/a | n/a | 0.66 (0.65-0.68) |
| 2 | n/a | ref | ref | ref |
| 3 | n/a | 1.37 (1.36-1.38) | 1.26 (1.23-1.28) | 1.27 (1.24-1.31) |
| 4 | n/a | 1.73 (1.71-1.75) | 1.53 (1.49-1.56) | 1.56 (1.51-1.60) |
| 5+ | n/a | 2.25 (2.20-2.29) | 2.02 (1.97-2.06) | 2.07 (2.01-2.13) |
| SES (socioeconomic status) | | | | |
| Low | 1.07 (1.06 - 1.07) | 1.04 (1.03 - 1.05) | 1.11 (1.09 - 1.12) | 1.02* (1.00 - 1.04) |
| High | ref | ref | ref | ref |
| Continuity: UPC index | 1.34 (1.33 - 1.35) | 1.08 (1.07 - 1.10) | 1.03 (1.00 - 1.06) | 0.60 (0.58 - 0.63) |
| Coordination: number of FPs | | | | |
| Saw < 5 FPs | 0.45 (0.45 - 0.46) | 0.67 (0.66 - 0.68) | 0.77 (0.75 - 0.78) | 0.73 (0.70 - 0.76) |
| Saw >= 5 FPs | ref | ref | ref | ref |
| Access: out-of-hours FP visit | | | | |
| Yes | 3.90 (3.86 - 3.93) | 2.48 (2.44 - 2.52) | 1.91 (1.86 - 1.95) | 1.95 (1.89 - 2.01) |
| No | ref | ref | ref | ref |
| *Note: * p ≥ 0.05, all other p < 0.05*  Table 2. Number and % of chronic conditions by population segment   \| **Population Segment** \| \| \| \| \| \| --- \| --- \| --- \| --- \| --- \| \| **Number of chronic conditions** \| **1 Low need**  **# (%)** \| **2 Multiple morbidities**  **# (%)** \| **3 Medically complex**  **# (%)** \| **4 Frail**  **# (%)** \| \| **0-1** \| 2,807,725(100.0%) \| 0 (0.0%) \| 39,676 (33.7%) \| 16,052 (24.4%) \| \| **2** \| 0 (0.0%) \| 289,820 (64.4%) \| 29,899 (25.4%) \| 15,515 (23.6%) \| \| **3** \| 0 (0.0%) \| 110,123 (24.5%) \| 21,622 (18.4%) \| 13,900 (21.1%) \| \| **4** \| 0 (0.0%) \| 37,436 (8.3%) \| 13,982 (11.9%) \| 9,821 (14.9%) \| \| **5+** \| 0 (0.0%) \| 12,818 (2.8%) \| 12,457 (10.6%) \| 10,547 (16.0%) \| \| **Total #** \| 2,807,725 \| 450,197 \| 117,636 \| 65,835 \| | | | | |
